# Development of a colloidal gold immunochromatographic assay utilizing dual-antibody sandwich method for detecting *Orientia tsutsugamushi*

**DOI:** 10.3389/fmicb.2024.1521015

**Published:** 2025-01-15

**Authors:** Qingyu Lu, Shiyin Yu, Sibo Wang, Min Cao, Liuxin Li, Miao Xin, Weilong Tan, Yong Qi, Yichen Lu, Xiaohui Xiong

**Affiliations:** ^1^College Food Science and Light Industry, Nanjing Tech University, Nanjing, China; ^2^Nanjing Bioengineering (Gene) Technology Center for Medicine, Nanjing, China

**Keywords:** *O. tsutsugamushi*, monoclonal antibody, polyclonal antibody, immunochromatography, colloidal gold

## Abstract

A colloidal gold immunochromatographic assay (ICA) based on a dual-antibody sandwich method was developed for the rapid and convenient detection of *Orientia tsutsugamushi* (*O. tsutsugamushi*) antigens in the early stages of infection. Monoclonal antibodies designed as 5B3 targeting the conserved region of 56 kDa outer membrane protein in various strains of *O. tsutsugamushi* were generated through cell fusion and screening techniques and combined with previously prepared polyclonal antibodies as detection antibodies to establish the ICA. Colloidal gold and polyclonal antibody-colloidal gold complexes were synthesized under optimized conditions. The nitrocellulose membrane was treated with 5B3 monoclonal antibody and goat anti-mouse antibody as the test and control lines, respectively. The ICA demonstrated robust sensitivity, with a minimum detection limit of 70.5 ng for the 56 kDa recombinant of the Gilliam strain. Furthermore, a detection limit of 1 × 10^6^ copies/μL DNA of *O. tsutsugamushi* was determined for both PT and SJ infected cell strains by constructing a relationship between cell number and copy number of the pathogen using a quantitative PCR-based standard curve. The assay also exhibited exceptional specificity, with no false positives observed against other bacterial species, including *Escherichia coli*, *Salmonella*, *Staphylococcus aureus*, and *Listeria monocytogenes*. In summary, an ICA which is sensitive, specific, and easy to operate was successfully established for the detection of *O. tsutsugamushi* in scrub typhus, potentially enabling early rapid point-of-care diagnosis of scrub typhus.

## 1 Introduction

Scrub typhus is an acute zoonotic febrile illness caused by *Orientia tsutsugamushi*, an obligate intracellular bacterium (Xu et al., 2017). This disease threatens approximately one billion people worldwide and causes nearly one million cases annually (Bhandari et al., 2022; John and Varghese, 2020). The clinical manifestations of scrub typhus closely resemble other acute febrile illnesses, such as murine typhus, malaria, dengue fever, and viral hemorrhagic fever. Moreover, it is not responsive to conventional antibiotics, leading to a high risk of misdiagnosis, delayed treatment, and potentially fatal outcomes (Ching et al., 2001). Therefore, accurate and timely diagnostic methods are crucial for the effective management of scrub typhus. Current diagnostic approaches for scrub typhus include pathogen isolation and culture, nucleic acid detection, and immunological assays. Blood samples from scrub typhus patients and organs or blood from infected animals (mainly rodents) can be used for pathogen isolation and culture. Various experimental animals, such as mice and chicken embryo yolk sacs, and established cell lines, including L929, HeLa, BHK21, and Vero, can be employed for the cultivation of *O. tsutsugamushi* (La Scola and Raoult, 1997; Tamura et al., 1995). Further identification is primarily performed through PCR amplification and gene sequencing of *O. tsutsugamushi*-specific genes. However, the culture conditions for *O. tsutsugamushi* are stringent, and its reproduction is slow, requiring an environment of 35°C and 5% CO_2_ for cell infection and growth (Luksameetanasan et al., 2007). Additionally, the isolation and culture of *O. tsutsugamushi* pose safety concerns and must be conducted in biosafety facilities (Paris and Dumler, 2016; Kim et al., 2006). Consequently, pathogen isolation is primarily used for laboratory research and is not suitable for routine diagnosis of scrub typhus due to its complex and time-consuming procedures (Luce-Fedrow et al., 2015).

Nucleic acid-based detection methods offer high specificity and sensitivity, enabling early detection of bacterial infections. Genes such as the 56 kDa type-specific antigen gene (Kumar et al., 2019), GroEL gene (Paris et al., 2009), 16S rRNA gene (Sonthayanon et al., 2009), TraD conjugative transfer protein gene (Prakash et al., 2022), and 47 kDa membrane protease gene (Jiang et al., 2004) are commonly used as target genes for specific nucleic acid detection of *O. tsutsugamushi*. Nucleic acid detection methods include polymerase chain reaction (PCR), multiplex PCR (mPCR), real-time fluorescence quantitative PCR (qPCR), loop-mediated isothermal amplification (LAMP), recombinase polymerase amplification, and electrochemical DNA sensors. Although these nucleic acid-based detection methods offer numerous advantages, their application is limited by the need for expensive equipment, specialized personnel, and the potential for contamination.

Immunological detection methods, such as the Weil–Felix reaction (Dasch et al., 1979), indirect fluorescent immunoassay (IFA) (Blacksell et al., 2015), enzyme-linked immunosorbent assay (ELISA) (Xue et al., 2019), and immunochromatographic assay (ICA) (Tang et al., 2022), are also widely used for scrub typhus diagnosis. The Weil-Felix reaction, despite its low specificity, can be employed for preliminary screening of scrub typhus in rural and grassroots areas with inadequate diagnostic facilities. Both ELISA and IFA have the advantages of sensitivity and specificity, but they lack standardized background controls for antibody detection. Therefore, research in different regions is required to determine their detection thresholds and distinguish patients from healthy individuals based on antibody background levels (Saraswati et al., 2019). IFA is recognized as the gold standard for the diagnosis of scrub typhus, but it requires expensive equipment and trained professionals, limiting its application in remote areas and epidemic settings. In recent years, the colloidal gold immunochromatography method has gained attention as a research hotspot for the diagnosis of scrub typhus due to its accuracy, convenience, and rapid results. However, most relevant studies focus on the detection of specific antibodies against *O. tsutsugamushi* (Kim et al., 2013; Cao et al., 2007; Kingston et al., 2015; Diao et al., 2017). Antigen detection offers an advantage in the early stages of the disease, as antigens typically appear in patient blood samples earlier than antibodies.

To date, the only report focusing on the detection of *O. tsutsugamushi* antigens is by Indrawattana et al. (2022), who prepared monoclonal and polyclonal antibodies after immunization with the recombinant protein of 60 kDa GroEL protein of *O. tsutsugamushi*. Subsequently, they employed a colloidal gold immunochromatography method to detect fresh blood samples from patients with febrile diseases, demonstrating a high accuracy rate and indicating its potential for early field diagnosis of scrub typhus.

In this study, we aimed to develop monoclonal antibodies targeting the conserve region of 56 kDa outer membrane protein of *O. tsutsugamushi* and combine them with previously prepared polyclonal antibodies (Zhao et al., 2024) as detection antibodies to establish an ICA for detecting *O. tsutsugamushi* antigens. The assembly strategy involved spraying the previously prepared rabbit polyclonal antibodies against the 56 kDa *O. tsutsugamushi* antigen onto the gold-labeled pad, coating the test line with monoclonal antibodies against the 56 kDa *O. tsutsugamushi* antigen, and coating the control line with goat anti-rabbit secondary antibodies. This successfully assembled colloidal gold immunochromatographic test strip can be used to detect *O. tsutsugamushi* antigens, aiming to provide a rapid and convenient diagnostic tool for the early stages of scrub typhus.

## 2 Materials and methods

### 2.1 Bacterial strains, cells, and experimental animals

The pET-28a plasmid, preserved in our laboratory, was used to express the 56 kDa protein of the *O. tsutsugamushi*. The myeloma cell line SP2/0 was also stored in our laboratory. The L929 cell line infected with the SJ and PT strains of *O. tsutsugamushi* was preserved in our laboratory. Female BALB/c mice (SPF grade), aged 6–8 weeks, were purchased from the animal center of SpeedBio Company [Animal Production License No. SCXK (Beijing) 2019-0010; Animal Use License No. SYXK (Beijing) 2019-0030]. All mice were housed in the animal experimental room of Nanjing Pharmaceutical Biotechnology (Gene) Technology Center under standard conditions: temperature (24 ± 0.5) °C, humidity (55 ± 5) %, and a 12/12-h light/dark cycle. The study was approved by the Experimental Animal Ethics Committee of Nanjing Pharmaceutical Biotechnology (Gene) Technology Center.

### 2.2 Expression and purification of the recombinant protein of the 56 kDa conserved region of *Orientia tsutsugamushi*

The amino acid sequences of the 56 kDa proteins from different strains of *Orientia*, including Gilliam, Karp, Kato, Sj, Pt, Kawasaki, and Young worl, were aligned using BioEdit software, and a conserved region spanning amino acids 170–400 was identified. Specific primer pairs were designed using Primer Premier 5: F: ATGGGTCGCGGATCCGAATTC AATCCTCAGCTTGATCATG; R: TCTCGAGTGCGGCCGCAAGCTTATCTTCTTCTTGTTGAGCAG. DNA extracted from SJ *Orientia*-infected cell lines was used as a template to amplify the 717 bp target fragment by PCR. The fragment was cloned into the *EcoR* I and *Hind* III restriction sites of the pET-28a plasmid vector to prepare the recombinant plasmid. After induction and expression, the recombinant protein was purified using QIAGEN Ni-NTA Agarose, and its activity was verified by Western blot and indirect ELISA.

### 2.3 Preparation, screening, and identification of monoclonal antibodies

The previously prepared recombinant outer membrane protein of the 56 kDa Gilliam strain of *Orientia* (Cao et al., 2007) was injected subcutaneously at multiple sites into three mice (6–8 weeks old), with 1 mouse serving as a negative control. For the initial immunization, Freund’s complete adjuvant was used as the emulsifier, and the immunization dose was 100 μg per mouse. A total of three immunizations were performed, with a 15-day interval between each. Except for the first immunization, which was administered subcutaneously at multiple sites, the other two immunizations were administered intraperitoneally using Freund’s incomplete adjuvant as the emulsifier. Fifteen days after the third immunization, blood was collected from the tails of the mice, and the serum titers were detected using an indirect ELISA method with the 56 kDa recombinant outer membrane protein of Gilliam strain as the coating antigen. Mice with high titers were selected for booster immunization 3 days before cell fusion, with an intraperitoneal injection of 100 μg of recombinant protein. After the booster immunization, the mice were euthanized, and immune splenocytes and myeloma cells were obtained for cell fusion. After 3–4 rounds of subcloning, stable monoclonal cell lines were obtained and expanded. Approximately 1 × 10^6^–5 × 10^6^ cells were inoculated into the abdomen of each mouse. The ascites were purified using Protein G affinity chromatography columns, and the titers of the purified ascites were detected using an indirect ELISA method.

Using the 56 kDa conserved region BS-717 recombinant protein as the screening antigen, hybridoma cell lines that specifically recognized the BS-717 protein were screened by indirect ELISA. The obtained monoclonal antibody (mAb) was further purified and identified by ELISA and Dot ELISA. Dot-ELISA was performed as follows: nitrocellulose (NC) membranes were cut into 1 × 1 cm squares and placed in a 24-well plate. Five microliters of recombinant protein, disrupted Pt-infected cell suspension, Sj-infected cell suspension, and L929 cell suspension were pipetted onto the center of the NC membranes, respectively, and dried at 37°C for 20 min. Five hundred microliters of blocking solution was added, and the membranes were oscillated at 37°C for 1 h for blocking. Afterward, the membranes were washed three times with PBST for 5 min each. The mAbs were diluted 1,000-fold in blocking solution and added to the 24-well plate at 200 μL/well. The plates were oscillated at 37°C for 1 h, followed by washing with PBST. Then, HRP-conjugated goat anti-mouse secondary antibody diluted 2000-fold was added to the wells at 200 μL/well and oscillated at 37°C for 1 h, followed by washing with PBST. After thorough washing, excess liquid was removed, and 20 μL of DAB color developing solution was pipetted onto the center of the NC membranes. The reactions were allowed to proceed in the dark for 10 min. After the reaction, the membranes were washed three times with distilled water, and excess liquid was removed. Brownish yellow spots on the membranes indicated positive results, while the absence of spots indicated negative results. The disrupted L929 cell supernatant served as a negative control, the 56 kDa recombinant protein served as a positive control, and the Pt and Sj-infected cell strains of *Orientia* served as detection antigens.

### 2.4 Preparation and characterization of colloidal gold

Colloidal gold was prepared using the sodium citrate reduction method of chloroauric acid. A rotor soaked in aqua regia was placed in a conical flask, and the rotation speed was adjusted to stir the liquid evenly. The final concentration of chloroauric acid was adjusted to 0.01% using ultrapure water. The chloroauric acid solution was brought to a boil, and the flask mouth was covered with aluminum foil. Sodium citrate was then added, and the mixture was heated for 30 min. During this process, the color changed from yellow to gray-black and finally to wine red. The solution was then cooled to room temperature. The colloidal gold solution was characterized using a UV–visible spectrophotometer (450–600 nm) and transmission electron microscopy (TEM).

### 2.5 Optimization and preparation of antibody-colloidal gold conjugates

To establish the optimal pH, the sodium chloride disruption method is employed. Eight 1.5 mL centrifuge tubes are prepared, each containing 1 mL of the prepared colloidal gold solution. The pH of these solutions is adjusted to 6.0, 6.5, 7.0, 7.5, 8.0, 8.5, and 9.0 using 0.1 M K_2_CO_3_, while the eighth tube containing the untreated colloidal gold solution serves as a blank control. An excess of polyclonal antibody is added to each tube, followed by vigorous shaking to ensure homogeneous mixing. The tubes are then allowed to stand at room temperature for 15 min. Next, 100 μL of 10% NaCl solution is added to each tube, and the mixtures are left undisturbed at room temperature for 2 h. During this period, the color of each solution is observed. The minimum pH that results in a solution color closest to the original color of the colloidal gold without any aggregation or precipitation is selected as the optimal pH. This optimal pH ensures the stability of the colloidal gold and promotes effective antibody binding, critical for the subsequent immunological reactions.

With adjusted optimum pH, eight 1.5 mL centrifuge tubes are prepared, each containing 1 mL of the prepared colloidal gold solution. The pH of the colloidal gold solution in each tube is adjusted to the optimal pH using 0.1 M K_2_CO_3_. Subsequently, 40, 35, 30, 25, 20, 15, 10, and 5 μg of polyclonal antibody are added to each tube, respectively. The mixtures are thoroughly mixed and allowed to stand at room temperature for 15 min. Following this, 100 μL of 10% NaCl solution is added to each tube, and the tubes are left undisturbed for 2 h. When an excessive amount of protein is added, the color of the solution remains unchanged. Conversely, when the protein amount is insufficient, the solution turns blue and aggregation occurs. Therefore, the protein concentration at the intersection of these two phenomena is selected. To ensure optimal stability and sensitivity, an additional 10% of this protein concentration is added, resulting in the determination of the optimal protein labeling amount.

The colloidal gold solution was adjusted to the optimal pH and stirred using a magnetic stirrer. The anti-Gilliam 56 kDa polyclonal antibody at the optimal protein concentration was added to the colloidal gold solution, and the mixture was stirred for 30 min using the magnetic stirrer. Subsequently, 1% PEG-20000 was added, and stirring was continued for another 30 min. Then, 10% BSA solution was added, and stirring was continued for 30 min to block non-specific binding sites. The solution was transferred to a centrifuge tube and stored overnight in a 4°C refrigerator. The next day, the solution was centrifuged at 12,000 rpm for 20 min, and the supernatant was discarded. The gold-labeled precipitate was dissolved in one-tenth of the original volume of resuspension solution and stored at 4°C.

### 2.6 Preparation of immunochromatographic test strip

**Figure 1:**
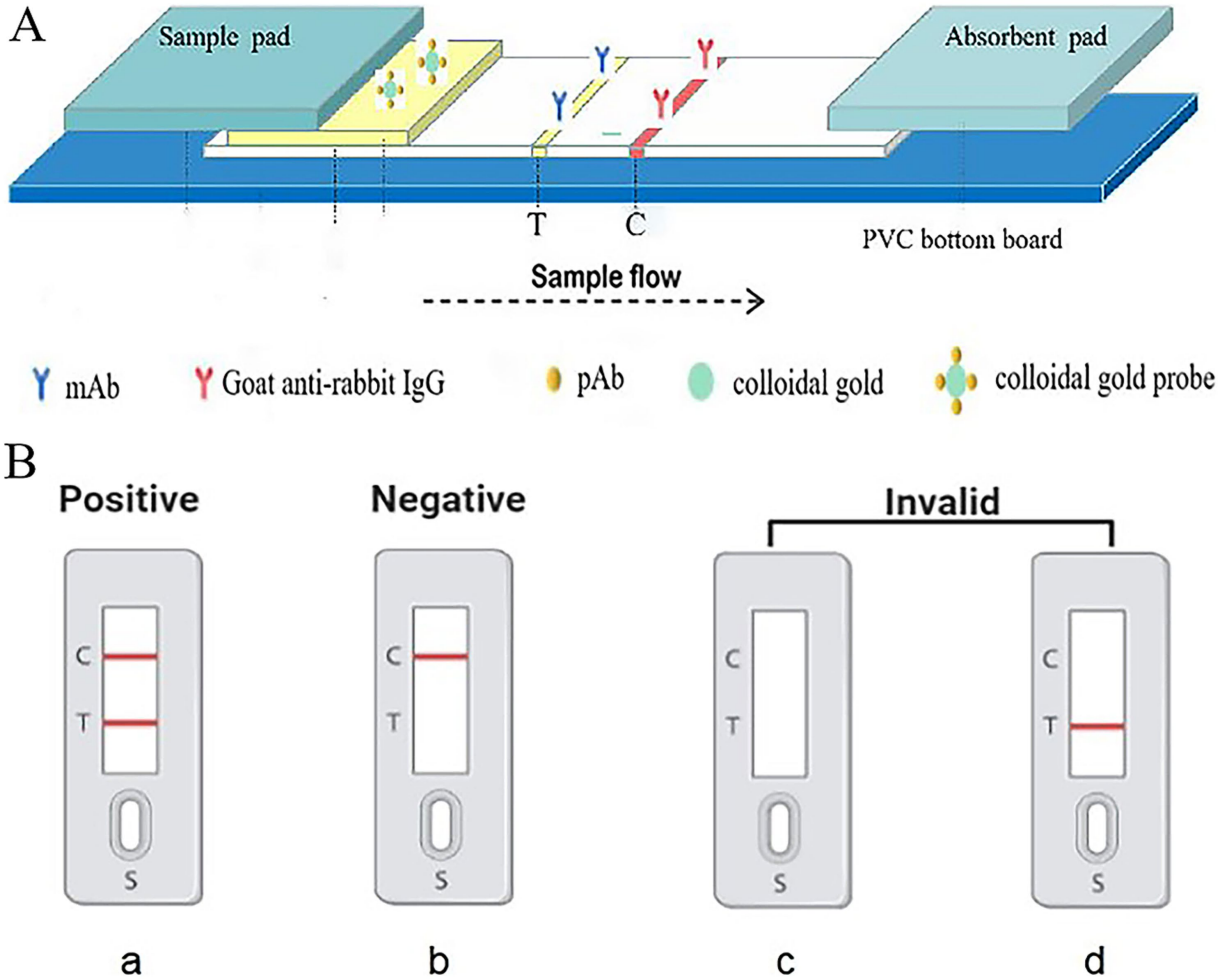
Schematic representation of the design of gold immunochromatographic assay strip. Anti-56 kDa monoclonal antibodies and goat anti-rabbit IgG were immobilized on a nitrocellulose (NC) membrane as the test (T) line and control (C) line, respectively, negative, positive, and invalid results.

Each 3 mm × 60 mm strip consists of five components: a polyvinyl chloride (PVC) support plate, a nitrocellulose (NC) membrane, an absorbent pad, and a conjugate pad (Figure 1A). The NC membrane is adhered to the center of the PVC support plate. An anti-56 kDa monoclonal antibody (0.5 mg/mL) is sprayed onto the test region (“T”) at a speed of 1 μL/cm, and goat anti-rabbit (0.5 mg/mL) is sprayed onto the control region (“C”) at the same speed using an automatic benchtop fine sprayer (Autokun, Hangzhou, China). These regions are located in the middle of the NC membrane, with a distance of 4 nm between them. The membrane is then dried at 37°C for 4 h, sealed in a plastic bag, and stored in a desiccator at room temperature (as shown in Figure 1A). The sample pad and absorbent pad are attached to one end of the PVC support plate, overlapping the NC membrane by 2 mm. The prepared strips are stored at room temperature in a plastic box containing silica gel desiccant.

### 2.7 Function of the ICA detection system

In the assay, 30 μL of sample solution is applied to the sample pad. Then, due to capillary action, the solution migrates through the nitrocellulose (NC) membrane towards the absorbent pad. When the liquid passes through the test line (T line), the *O. tsutsugamushi* in the sample solution that has already bound to the colloidal gold anti-56 kDa polyclonal antibody is fixed on the membrane by the anti-56 kDa monoclonal antibody. The liquid continues to migrate to the control line (C line), where the colloidal gold anti-56 kDa polyclonal antibody is fixed by goat anti-rabbit IgG. After approximately 15 min, the presence of red bands on both the T and C lines can be observed visually. As shown in the Figure 1B, the presence of two red bands indicates a positive result for scrub typhus. The absence of a red band at the T line indicates a negative result. The C line, which serves as a control for the assay, must show a red band regardless of whether the T line is red or not; if there is no red band on the C line, the test is considered invalid.

### 2.8 Detecting *Orientia tsutsugamushi* in infected cells using test strip

As *O. tsutsugamushi* is an intracellular parasitic bacterium, its cultivation and isolation process being time-consuming and complex, making it extremely difficult to count and quantify. This study aims to construct a standard curve using qPCR and then establish a relationship between the number of infected cells and the copy number of *O. tsutsugamushi* based on this standard curve, ultimately determining the detection limit of the colloidal gold immunochromatographic test strip.

Establishment of a qPCR standard curve for *O. tsutsugamushi*. A recombinant plasmid containing the 56 kDa fragment of *O. tsutsugamushi*, preserved in our laboratory, was used to calculate its copy number and then subjected to a 10-fold gradient dilution, with eight dilutions (10^−1^ to 10^−8^) serving as templates for qPCR. Six parallel reactions were performed for each gradient, qPCR was conducted under the conditions specified in Supplementary Table S3. The qPCR amplification was performed using primers Ot-F: 5′-GGAGGTGAGATAAAGGC-3′ and Ot-R: 5′-ATAGTCAATACCAGCACAA-3′. The reaction data were read using LightCycler^®^ 96 SW to determine the linear relationship.

Establishment of the relationship between *O. tsutsugamushi* copy number and cell count. Sj and Pt cells were cultivated until the cell culture flask was completely covered. All cells were scraped off and centrifuged at 1,500 rpm for 5 min. The supernatant was discarded, and the cells were resuspended in 1 mL of PBS, 20 μL of the suspension was taken for observation and counting using a hemocytometer. A 10-fold gradient dilution of the counted cells was performed with PBS for DNA extraction. qPCR was conducted on the extracted DNA, and the relationship between cell count and copy number was determined based on the standard curve, enabling quantitative analysis of the number of *O. tsutsugamushi* cells.

### 2.9 Detection limit of colloidal gold immunochromatographic test strip

#### 2.9.1 Detection limit of recombinant protein using test strip

The 56 kDa recombinant protein was diluted in a 2-fold serial dilution using sterile PBS, and 30 μL of each dilution was applied to the sample pad.

#### 2.9.2 Detection limit of *Orientia tsutsugamushi* in infected cells using test strip

Sj and Pt cells were collected by centrifugation at 1,500 rpm for 5 min, resuspended in 1 mL of PBS, and 20 μL of the cell suspension was taken for counting. Glass beads (2.5 mm) were added to the remaining cells, placed on a shaker for 30 s, and then immersed in crushed ice for 30 s. This process was repeated 15 times to disrupt the cells and release *O. tsutsugamushi*. The disrupted cell suspension was transferred to a clean centrifuge tube, and cell debris was removed by centrifugation at 2,000 rpm for 10 min. A 10-fold gradient dilution of the supernatant sample was performed. Add the diluted sample to the sample pad, react for 15 min, and then observe the results of the test strip.

### 2.10 Specificity and stability of colloidal gold immunochromatographic test strips

The specificity of colloidal gold immunochromatographic test strips was evaluated using *Escherichia coli*, *Salmonella*, *Staphylococcus aureus*, and *Listeria monocytogenes*.

To determine the stability of the colloidal gold immunochromatographic test strips, they were sealed and stored at room temperature for 7 days, 1 month, 2 months, and 3 months, respectively. At each time point, the test strips were retrieved, and their stability was verified using samples.

### 2.11 Statistical analysis

Each experiment was performed with three replications. Data were analyzed by a two-tailed, unpaired *t*-test. A *p*-value of <0.01 was considered highly significant.

## 3 Results

### 3.1 Screening, expression, and identification of the recombinant protein of the conserved region of the 56 kDa protein

**Figure 2:**
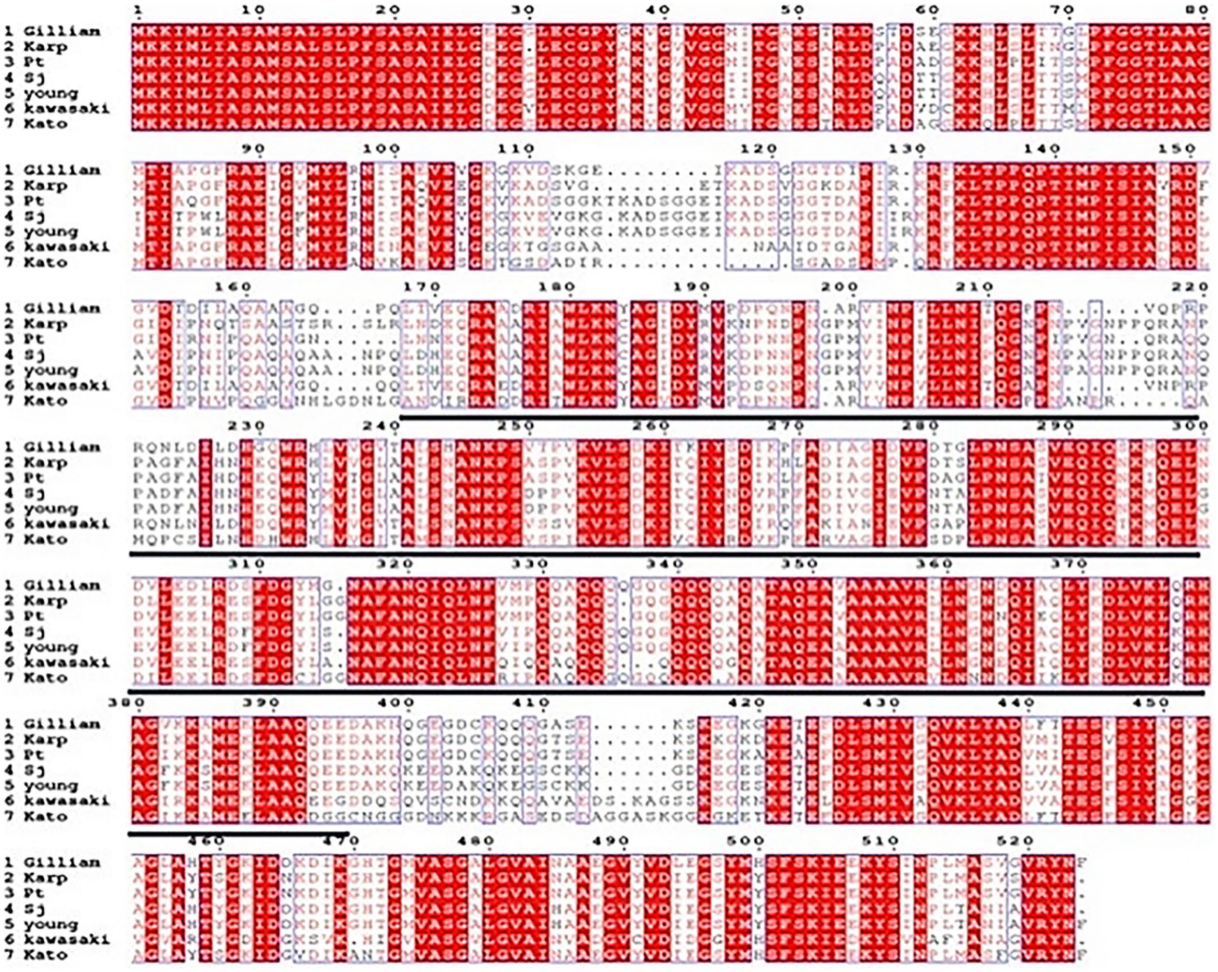
Alignment analysis of the amino acid sequences of the 56 kDa protein of various strains of *O. tsutsugamushi*.

The amino acid sequences of the 56 kDa protein from the prevalent standard strains and local strains of *O. tsutsugamushi* in China were aligned using ESPript 3.0 software, and the results are shown in Figure 2. The amino acid sequence within the region marked by a black line (170–400) was selected as the target protein for experimentation. The gene encoding this regional protein was cloned into the pET-28a plasmid to obtain the recombinant plasmid. After induction and expression, SDS-PAGE analysis revealed a 32 kDa target band (Supplementary Figures S1A,B). Western blot results (see Supplementary Figures S1C,D) and ELISA results (see Supplementary Table S1) indicated that the recombinant protein specifically reacted with sera from patients with scrub typhus caused by *O. tsutsugamushi* but did not react with negative sera, suggesting that this conserved protein possesses biological activity.

### 3.2 Screening, purification, and binding activity identification of monoclonal antibodies

After the cell fusion process, six hybridoma cell lines secreting antibodies against the Gilliam 56 kDa protein were successfully isolated through four rounds of subcloning, followed by purification and identification procedures (refer to Supplementary Figure S2 for details). To further refine the selection, a conservative region protein, Sj-717, was employed in an ELISA-based screening method to identify a specific hybridoma cell, designated as 5B3, capable of secreting antibodies that specifically recognized this conserved region (Supplementary Figure S3). The 5B3 was furtherly purified (Supplementary Figure S4) and identified (Supplementary Table S2).

**Figure 3:**
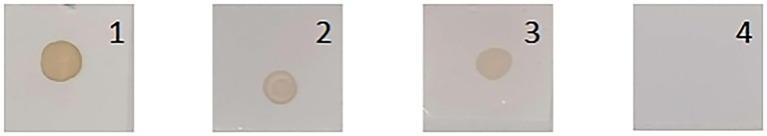
Identification of mAb 5B3 by Dot ELISA. 1: Positive, BS-717 recombinant protein. 2: Pt infected cell lines. 3: Sj infected cell lines. 4: Negative, L929 cell.

Subsequently, the binding capability of the 5B3 monoclonal antibody (mAb) towards *Orientia* strains was rigorously evaluated using Dot ELISA. The experimental outcomes, as presented in Figure 3, reveal that upon utilizing the 5B3 mAb to detect Pt and Sj *Orientia*-infected cell lines, distinct yellowish-brown spots emerged on the nitrocellulose membrane (NC), mirroring the positive control pattern. Conversely, no such bands were observed in the negative control, confirming that the 5B3 mAb exhibits robust reactivity towards *Orientia*-infected strains. This discovery underscores the potential of the 5B3 mAb as a valuable diagnostic tool for the development of rapid immunological detection methods.

### 3.3 Preparation and characterization of colloidal gold

**Figure 4:**
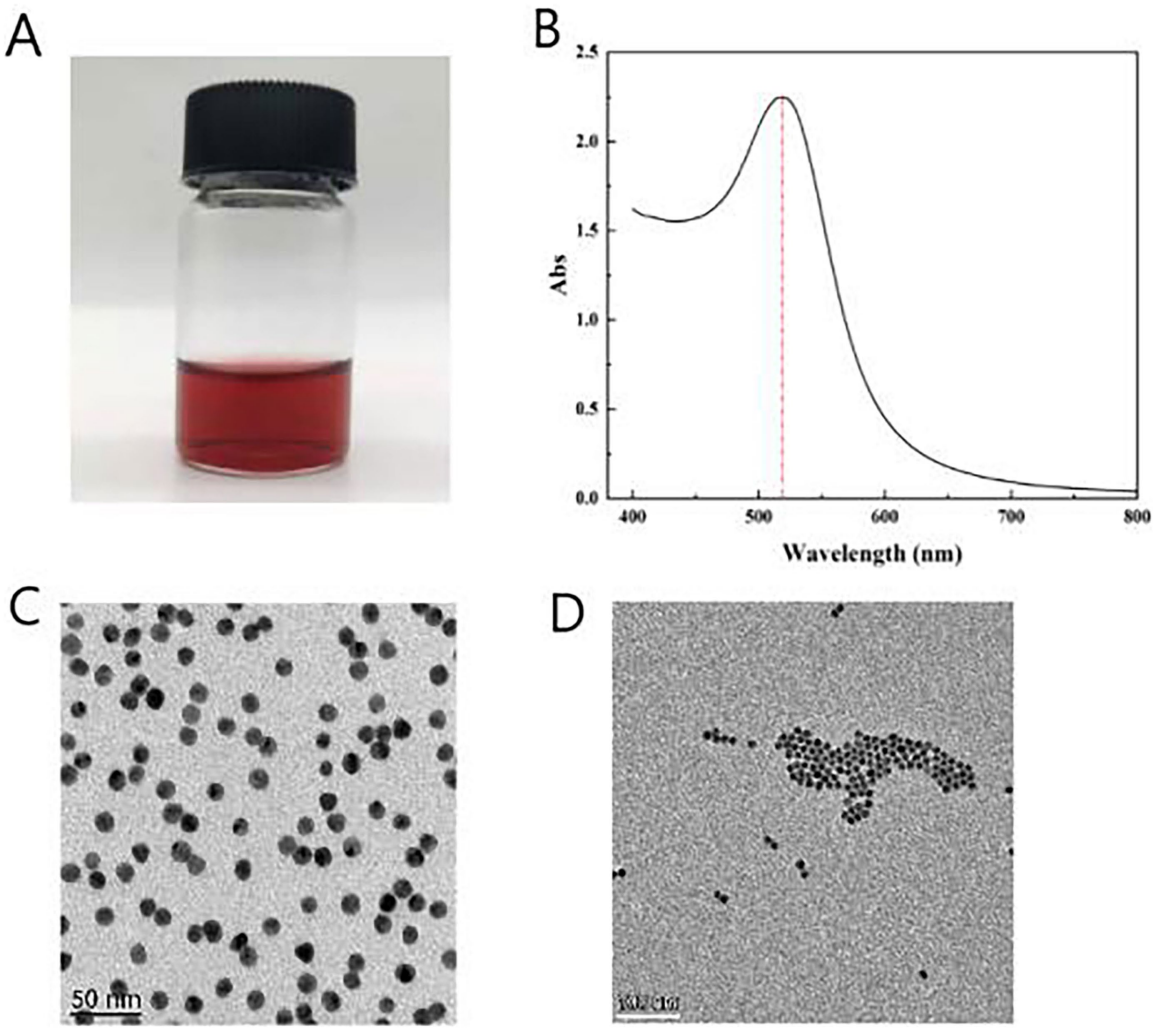
Synthesis of colloidal gold solution. **(A)** Color of colloidal gold solution (after adding 2 mL of 1% trisodium citrate). **(B)** Absorption peak obtained from UV full-wavelength scanning. **(C)** TEM images of colloidal gold solution 50 nm scale. **(D)** TEM images of colloidal gold solution 100 nm scale.

The size and uniformity of colloidal gold particles significantly impact the sensitivity of assays (Frens, 1973). In this study, a colloidal gold solution was prepared using the citrate trisodium reduction method of chloroauric acid, resulting in a clear, transparent, and wine-red solution without any suspended particles or precipitates, consistent with the expected wine-red color (Figure 4A). The particle size distribution of the colloidal gold can be preliminarily evaluated using ultraviolet (UV) full-wavelength scanning. A higher absorption peak indicates larger particle sizes, while a smaller peak width-to-height ratio signifies better size uniformity. The UV-visible spectrophotometer was employed to detect the maximum absorption peak, which was found to be at 521 nm, with a relatively narrow peak width, indicating the uniformity of the synthesized colloidal gold particles (Figure 4B).

Transmission electron microscopy (TEM) scanning revealed well-formed, spherical colloidal gold particles without overlapping or aggregation (Figures 4C,D). The particle size was measured using ImageJ software, confirming an average diameter of 20 nm. The prepared gold particles exhibited uniform size and excellent dispersion, making them suitable for specific detection applications.

### 3.4 Optimization of ICA experimental parameters

#### 3.4.1 Optimization of pH for labeling colloidal gold solution

Colloidal gold particles can bind to proteins through electrostatic adsorption at an appropriate pH, and this binding method does not affect the properties of the protein (Boulos et al., 2013). When the pH is too low, it will disrupt the electrostatic charge of the colloidal gold particles, causing them to aggregate and form black precipitates after standing; when the pH is too high, the amount of protein adsorbed by the colloidal gold will decrease, affecting the sensitivity of the test strip. Therefore, it is necessary to select an appropriate pH for labeling proteins.

**Figure 5:**
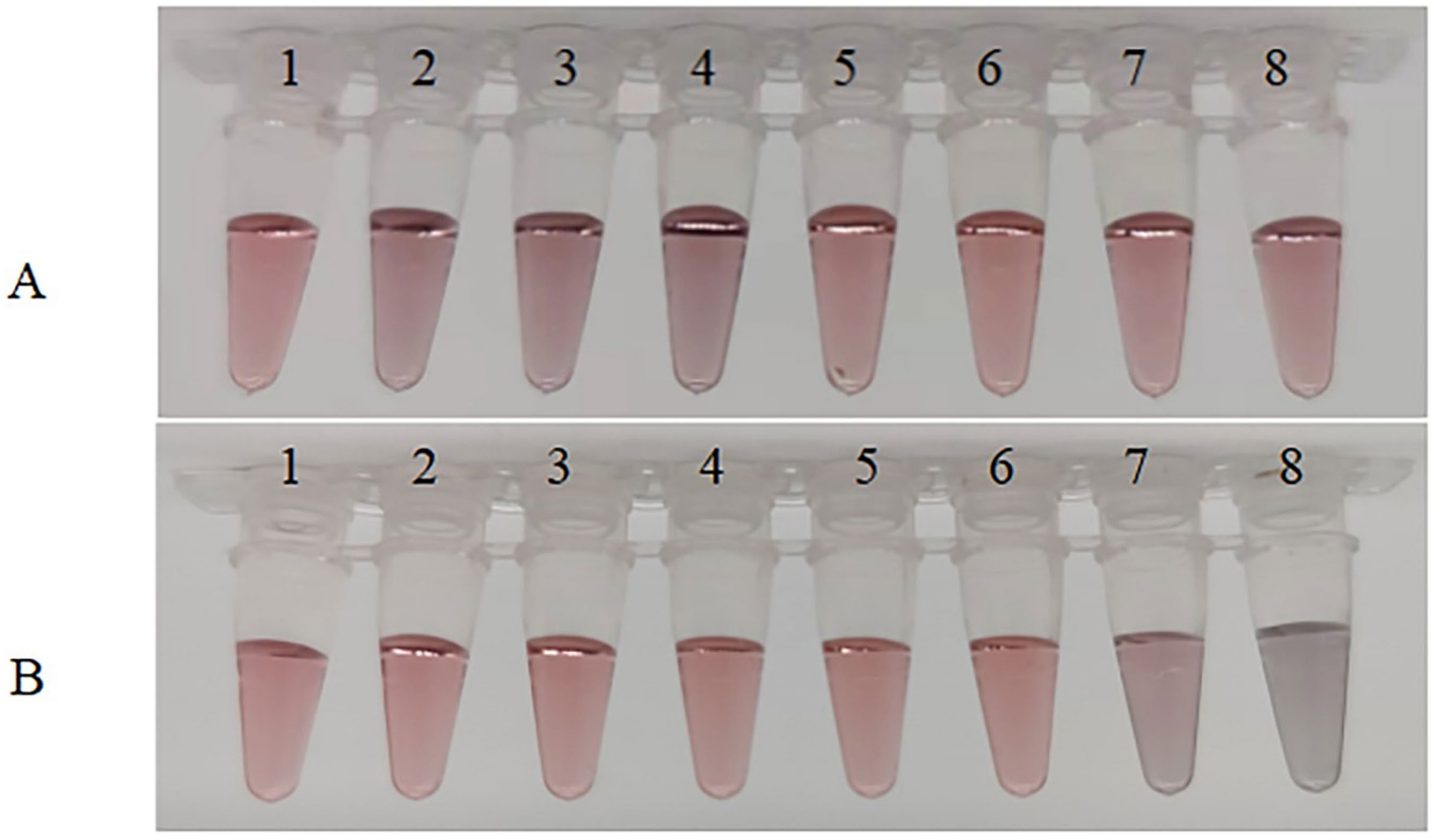
Optimization of experimental parameters. **(A)** Optimization of pH of colloidal solution. The pH of the colloidal solution was adjusted by adding different volumes of 0.1 mol/L K_2_CO_3_. Sample 1 serves as a blank control, while samples 2–8 have pH values of 6.5, 7.0, 7.5, 8.0, 8.5, 9.0, and 9.5, respectively. **(B)** Optimization of the amount of anti-56 kDa polyclonal antibody for coupling with colloidal gold solution. Under optimal pH conditions, different amounts of polyclonal antibodies were added to the colloidal gold solution. The antibody concentrations in samples 1–8 were 40, 35, 30, 25, 20, 15, 10, and 5 μg/mL, respectively.

**Figure 6:**
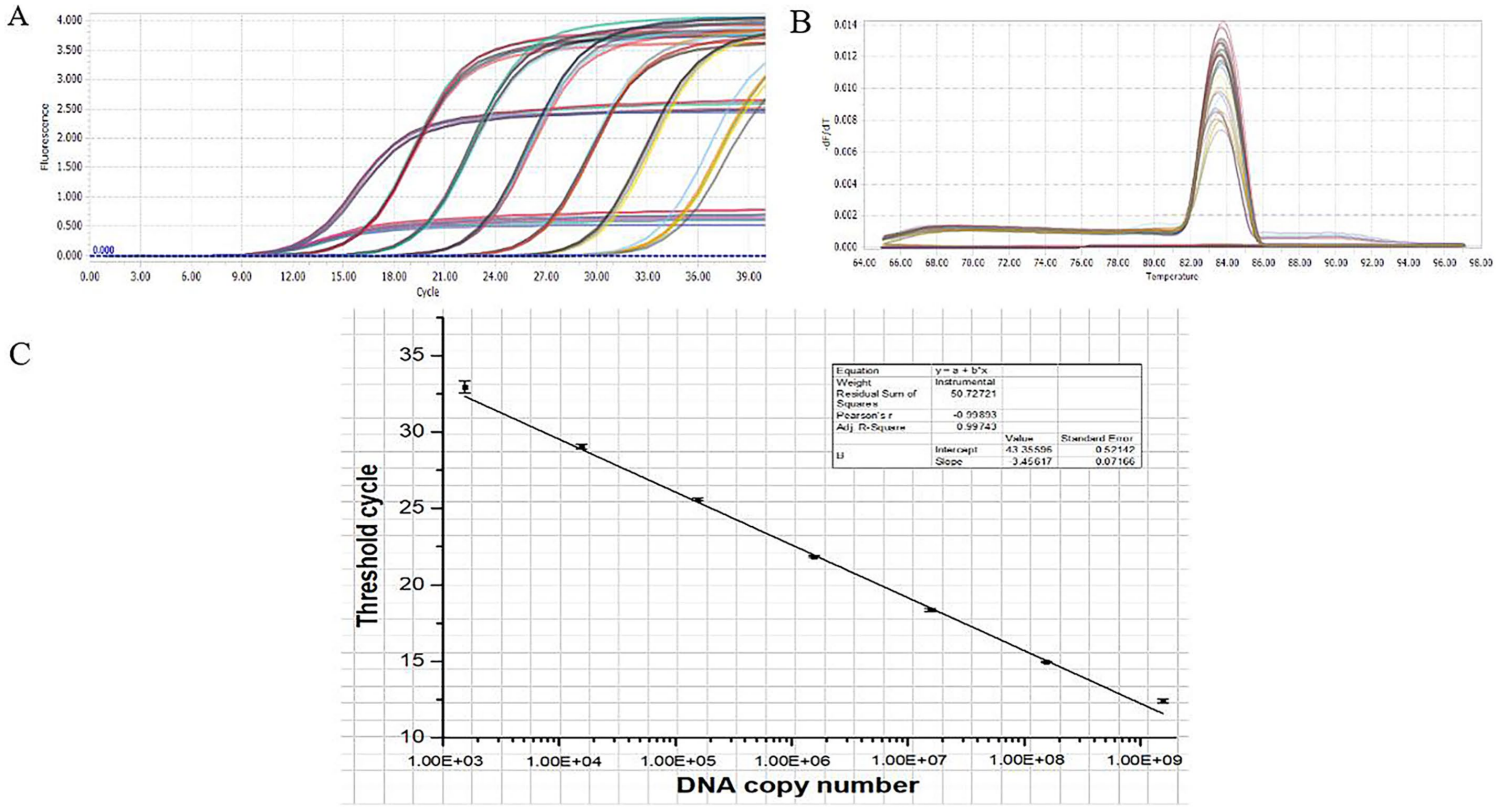
qPCR standard curve. **(A)** qPCR amplification curve. **(B)** qPCR melting curve. **(C)** qPCR standard curve.

As can be seen from Figure 5A, when the pH is lower than 7.5, precipitation occurs, causing the product to adhere to the walls of the centrifuge tube, and the solution appears black or purple to the naked eye (Figure 5A-2–4); when the pH is 8.0 or 8.5, the color of the solution is very similar to that of the reference colloidal gold solution. Always appearing as a burgundy color and remaining stable (Figure 5A-5, 6). Additionally, a pH of 8.5 is close to the isoelectric point of the anti-56 kDa polyclonal antibody, which maximizes the adsorption between the anti-56 kDa polyclonal antibody and the colloidal gold, promoting the formation of a stable gold-labeled antibody solution. When the pH is too high, the amount of protein adsorbed by the colloidal gold decreases, affecting the sensitivity of the test strip. Therefore, the optimal pH for preparing colloidal gold-labeled polyclonal antibodies is 8.5 (Figure 5A-6).

#### 3.4.2 Optimization of the content of labeled polyclonal antibodies

When using colloidal gold to label proteins, using too little labeled protein can result in excess gold particles that are not coated, while using too much protein can be wasteful. Therefore, it is necessary to select an appropriate amount of protein. As shown in Figure 5B, in the first six tubes, the solution appears as a clear and transparent burgundy color due to sufficient protein in the colloidal gold solution, indicating that the protein amount is adequate. However, the color in the seventh tube is slightly blue, and the eighth tube is completely blue-black, indicating that the protein amount in these two tubes is too low. Therefore, the protein amount in the sixth tube is determined as the critical value, with a stable minimum concentration of 15 μg/mL. Thus, the optimal protein amount is 20 μg/mL.

### 3.5 Detecting *Orientia tsutsugamushi* in infected cells using test strip

#### 3.5.1 Establishment of a qPCR standard curve for *Orientia tsutsugamushi*

The results of the qPCR amplification for *O. tsutsugamushi* are presented in Figures 6A,B. The amplification curves exhibited a characteristic S-shaped pattern, with the cycle threshold (Ct) values increasing as the template concentration decreased, indicating clear gradients (Figure 6A). The data within each group showed good parallelism, and the melting curve exhibited a single peak (Figure 6B), suggesting good primer specificity, correct qPCR amplification products, and reliable data. Figure 6C illustrates the standard curve established based on the linear relationship between copy numbers and Ct values, with a linear correlation coefficient of 0.99743, indicating a strong linear correlation between copy numbers and Ct values.

#### 3.5.2 Establishment of the relationship between *Orientia tsutsugamushi* DNA copy number and cell count

The Sj and Pt infected cells were diluted and counted separately. DNA was extracted from the diluted cells for qPCR analysis. Based on the established qPCR standard curve, the relationship between cell number and copy number was further determined. The specific corresponding numerical relationship is shown in Table 1.

**Table 1:** Relation between cell number and DNA copy number of *O. tsutsugamushi*.

Cell number	DNA copy number (copies/μL)
3 × 10^6^	1 × 10^8^
3 × 10^5^	1 × 10^7^
3 × 10^4^	1 × 10^6^
3 × 10^3^	1 × 10^5^
3 × 10^2^	1 × 10^4^
3 × 10^1^	1 × 10^3^

### 3.6 Determination of the detection limit of the test strip

#### 3.6.1 Detection limit of the test strip for recombinant protein

**Figure 7:**
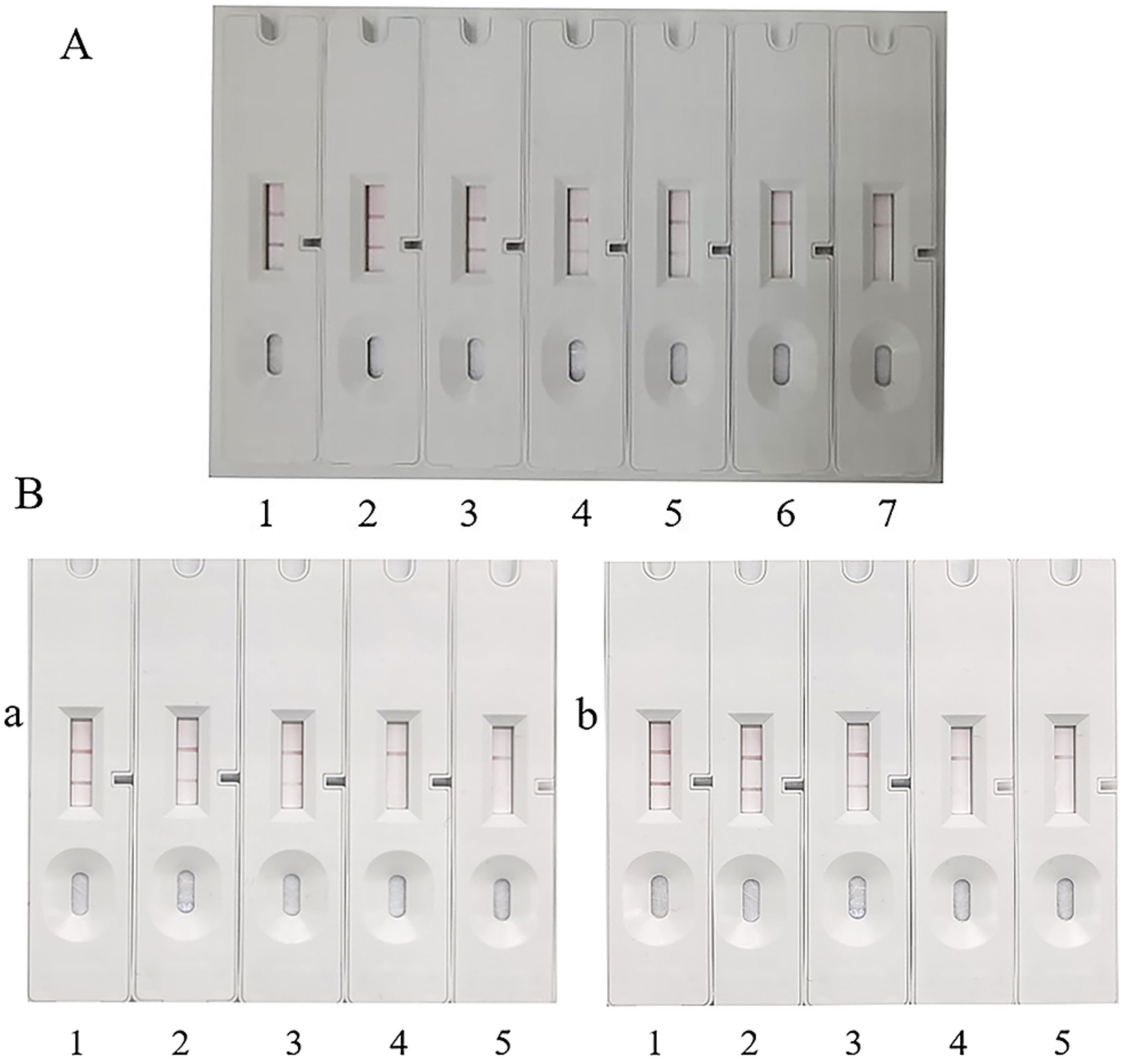
Detection limit of ICA. **(A)** Detection limit of ICA for recombinant protein. 1: 37.00 μg/mL. 2: 18.75 μg/mL. 3: 9.38 μg/mL. 4: 4.69 μg/mL. 5: 2.35 μg/mL. 6: 1.18 μg/mL. 7: Blank control, PBS. **(B)** Detection limit of ICA for *O. tsutsugamushi* DNA. 1: 1 × 10^8^ copies/μL. 2: 1 × 10^7^ copies/μL. 3: 1 × 10^6^ copies/μL. 4: 1 × 10^5^ copies/μL. 5: Blank control, PBS. **(a)** Sj cell test. **(b)** Pt cell test.

To determine the limit of detection (LOD) of the assay method, the 56 kDa recombinant protein was serially diluted, and 30 μL of each dilution was applied to the sample pad. As shown in Figure 7A-1–6, the color intensity of the T-line decreased with the reduction of the concentration of the 56 kDa recombinant protein. At a concentration of 2.35 μg/mL, the T-line band was still visible (Figure 7A-5), albeit with relatively faint color intensity. However, when the concentration of the recombinant protein was reduced to 1.18 μg/mL, the T-line band of the test strip became invisible (Figure 7A-6). Repeated experiments consistently indicated that both the control (C) and test (T) lines were visible at a concentration of 2.35 μg/mL. No background signals were observed on the test strip under any test conditions. Therefore, the lowest detection limit of the test strip was determined to be 2.35 μg/mL. Given the sample volume of 30 μL, the actual minimum detectable amount was 70.5 ng.

#### 3.6.2 Determination of the detection limit of *Orientia* in cells by test strip

**Figure 8:**
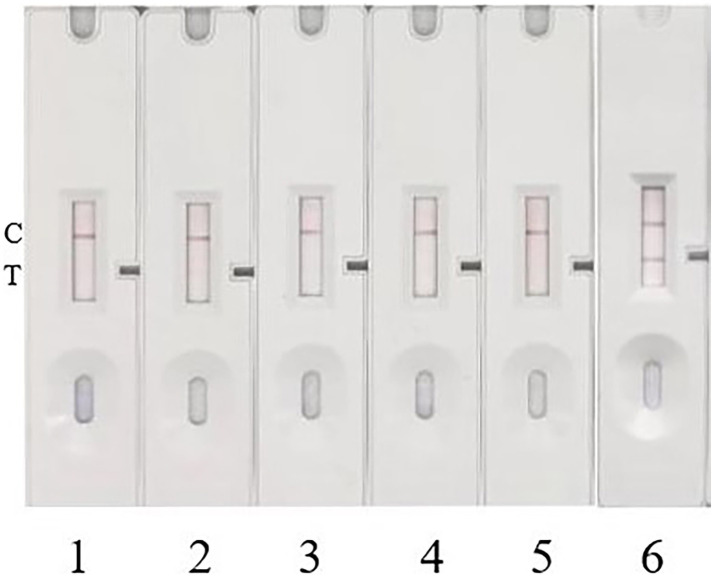
Specificity test. 1, 2, 3, and 4 represent *Escherichia coli*, *Salmonella*, *Staphylococcus aureus*, and *Listeria monocytogenes*, respectively. 5 serves as a blank control with sterile trypticase soy broth (TSB) culture medium, and 6 serves as a positive control with Pt-infected strain.

The number of infected cells was adjusted to 3 × 10^6^, 3 × 10^5^, 3 × 10^4^, 3 × 10^3^, 3 × 10^2^, and 3 × 10^1^, respectively. After cell lysis, the released *O. tsutsugamushi* was applied to the sample pad, and PBS was used as a blank control. The experimental results are shown in Figure 8. Sj cells diluted to 1 × 10^6^ still showed positive bands (Figure 7B-a-3), and Pt cells diluted to 1 × 10^6^ were also detected as positive (Figure 7B-b-3). Referring to the qPCR results and the standard curve, the minimum detection limit of *O. tsutsugamushi* cells was determined to be 1 × 10^6^ copies/μL DNA.

### 3.7 Specificity and stability of ICA test strip

To investigate the specificity of this method, four common pathogenic bacteria, including *Escherichia coli*, *Salmonella*, *Staphylococcus aureus*, and *Listeria monocytogenes*, were cultured to a concentration of 1 × 10^7^ CFU/mL. The samples were then added to the prepared colloidal gold immunochromatographic test strip for detection. Repeated experimental results showed that no bands appeared on the T-line of the test strip for all four pathogenic bacteria, indicating negative detection results for these pathogens (Figure 8). This demonstrates that the prepared colloidal gold test strip detection method has good specificity. The test strips, which can be stored at room temperature for up to 10 months, showed consistent results with previous experiments after equilibrating for 30 min and applying the sample to the sample pad, indicating good stability of the test strips.

## 4 Discussion

The ICA is a novel detection technology that combines the principles of immunogold labeling and chromatography. Due to its advantages of portability, high sensitivity, ease of operation without the need for sophisticated laboratory instruments, and visible results, ICA has been widely applied to the rapid detection of various pathogens, including parasites, viruses, and bacteria. In the diagnostic research of scrub typhus, most ICA methods have focused on the detection of specific antibodies against *O. tsutsugamushi*. For instance, Kim et al. (2013) developed a ICA method to detect corresponding antibodies in blood samples using a mixture of antigens from five different strains of Karp, Kato, Gilliam, Boryong, and Kangwon as diagnostic antigens. Kingston et al. (2015) utilized the InBios rapid test kit to detect antibodies against *O. tsutsugamushi* in the blood of febrile patients in India and Thailand. Cao et al. (2007) conjugated colloidal gold with truncated recombinant antigens of 56 kDa from the prevalent Ptan strain and Gilliam strain in China, preparing test strips capable of detecting specific total antibodies, IgM, and IgG against scrub typhus in blood samples. According to reports, a GICA-based detection method for scrub typhus exhibited good sensitivity and specificity (96.8 and 93.3%, respectively) in detecting IgM antibodies, while the specificity of total antibody detection was relatively poor in the same study (Blacksell et al., 2010). Diao et al. (2017) found that the sensitivity and specificity of IgM detection using ICA (98.6 and 98.2%, respectively) were higher than those of IgG (97.1 and 97.7%, respectively), with no cross-reactivity with other diseases. These ICA methods for detecting antibodies in blood samples are rapid and simple, making them suitable for detecting antibodies produced in the later stages of the acute phase of the disease. Additionally, it is essential to consider the background of normal serum antibodies and the serotypes of *O. tsutsugamushi* in the local area. Since antigens typically appear earlier than antibodies in patient blood samples, antigen detection offers an advantage in the early stages of the disease. However, as *O. tsutsugamushi* is an intracellular parasitic bacterium, the detection process is complex, and obtaining relevant specific antibodies is challenging, leading to limited research reports in this area.

The study conducted by the Indrawattana et al. (2022) is currently the only reported instance of utilizing the ICA method to detect *O. tsutsugamushi* antigens in scrub typhus. They developed an ICA kit by employing the 60 kDa GroEL protein of scrub typhus as the immunogen to produce monoclonal and polyclonal antibodies. Our research follows a similar technical route but chooses the 56 kDa protein of *O. tsutsugamushi* as the immunogen. The 56 kDa protein is the primary outer membrane protein of *O. tsutsugamushi* containing both conserved and variable regions. The conserved region sequences are highly homologous among the 56 kDa proteins of different *Orientia* serotypes while variable domains within their 56 kDa proteins allowing for the classification of *O. tsutsugamushi* into multiple serotypes. As a result, the 56 kDa outer membrane protein of *O. tsutsugamushi* is the most commonly used target antigen in immunological detection methods due to its potent immunogenicity, which elicits a robust humoral immune response in the host (Chi et al., 1997). This protein plays a crucial role in the diagnosis of scrub typhus. In our study, we utilize two kinds of antibodies including an anti-*Orientia* 56 kDa protein polyclonal antibody and an anti-*Orientia* conserved 56 kDa protein monoclonal antibody. The polyclonal antibodies were previously prepared by immunized the rabbit with 56 kDa protein of *O. tsutsugamushi*. The monoclonal antibody 5B3 was obtained by firstly immunized the mice with 56 kDa protein antigen and followed by screening with a highly conserved 56 kDa outer membrane protein which shared >99% homology among various strains of *O. tsutsugamushi*. Monoclonal antibodies exhibit excellent specificity but can be prone to false negatives due to their narrow reactivity against a single epitope. On the other hand, polyclonal antibodies, while not as specific as monoclonal antibodies, offer high antibody titers, sensitivity, and cost-effectiveness. Previous pathogen diagnostic studies have often used a combination of both types of antibodies, demonstrating that this approach can effectively enhance sensitivity and specificity (Ye et al., 2023; Song et al., 2022). Therefore, by leveraging both polyclonal and monoclonal antibodies against the 56 kDa protein of *O. tsutsugamushi*, we aim to improve the diagnostic accuracy for scrub typhus.

Detection sensitivity, often reflected by the limit of detection (LOD), is a crucial indicator for evaluating diagnostic reagents. Using the 56 kDa outer membrane recombinant protein as the target antigen, the LOD of our ICA strip was determined to be 70.05 ng. In contrast, the ICA kit developed by the Indrawattana et al. (2022) for a similar study could detect as low as 125 ng of GroEL chaperonin protein. However, determining the LOD of diagnostic kits using recombinant proteins in actual samples poses limitations, as *O. tsutsugamushi*, a predominantly intracellular parasite found in the buffy coat of blood samples, is difficult to culture, purify, quantify, and enumerate. Consequently, establishing the LOD of immunological diagnostic methods for *O. tsutsugamushi* remains a challenge, with the only reported study on ICA detection antigens failing to address this issue (Indrawattana et al., 2022). Unlike other model organisms such as *Escherichia coli*, where bacterial copy numbers can be directly assessed using optical density (OD) values, intracellular obligate parasites like *O. tsutsugamushi* cannot be similarly evaluated. Plaque purification methods, though capable of counting, are cumbersome, time-consuming, and impractical for large numbers (Moree and Hanson, 1992; Weinberg et al., 1969). Microscopic counting using Giemsa staining may also be attempted, but it suffers from poor accuracy and reproducibility (Hanson, 1991). Real-time quantitative PCR (qPCR) offers a solution by enabling the calculation of bacterial copy numbers. Giengkam et al. (2015) employed qPCR, leveraging a standard curve based on the copy number of the 47 kDa truncated gene, a single-copy gene in *O. tsutsugamushi*, to represent the bacterial load.

Inspired by this approach, our study ingeniously utilized qPCR to quantify *O. tsutsugamushi*. Firstly, a standard curve relating cycle threshold (Ct) to copy number was constructed by gradient diluting a single-copy 56 kDa protein clone plasmid. Subsequently, DNA extracted from gradient-diluted *O. tsutsugamushi*-infected cells was used as a template for fluorescence-based quantitative PCR amplification of the truncated 56 kDa outer membrane protein gene. Based on this standard curve, a relationship between cell count and *O. tsutsugamushi* copy number was established, ultimately enabling the determination of the LOD. Experimental results revealed that the LOD for both the Pt and Sj strains of the test strip was 1 × 10^6^ copies/μL. Specificity tests demonstrated no reaction with *Escherichia coli*, *Salmonella*, *Staphylococcus aureus*, or *Listeria monocytogenes*. Although limitations in resources prevented the use of strains closely related to *O. tsutsugamushi*, such as those causing spotted fever, as controls, the screening antigen used in this study for monoclonal antibody selection was highly conserved among different strains of *O. tsutsugamushi* and highly specific between species. This allowed for the selection of monoclonal antibodies that recognized epitopes within the conserved region of the 56 kDa protein, making them both broad-spectrum diagnostic antibodies targeting multiple serotypes of *O. tsutsugamushi* and specific diagnostic antibodies exclusive to *O. tsutsugamushi*, thereby ensuring the specificity and sensitivity of the entire reaction. After storing the test strips at room temperature for 6 months, samples were applied to the sample pad, and the results were consistent with previous experiments, indicating good stability of the prepared test strips. A limitation of this study is the lack of fresh clinical samples (such as patient serum and plasma) for sensitivity experiments. It cannot provide a testable hypothesis without evaluating the system on the natural samples. The complex composition of serum and plasma samples may affect result interpretation in practical applications. Since prolonged storage of samples can lead to autolysis of *O. tsutsugamushi*, the next step is to collect fresh samples during the epidemic season of scrub typhus in endemic areas to further evaluate the test strip’s detection performance.

In summary, this study aimed to develop monoclonal antibodies against the 56 kDa outer membrane protein of the Gilliam strain of *O. tsutsugamushi*. By combining these monoclonal antibodies with previously prepared polyclonal antibodies, a colloidal gold immunochromatographic assay (ICA) was established for the detection of *O. tsutsugamushi* in scrub typhus using the principle of the double antibody sandwich method. This method is sensitive, specific, and easy to operate, providing visible results within 15 min, indicating its promising application prospects in primary hospitals, remote towns, and epidemic outbreak areas.

## Data Availability

The original contributions presented in the study are included in the article/Supplementary material, further inquiries can be directed to the corresponding authors.
