# Supplementary material for: Development of a colloidal gold immunochromatographic assay utilizing dual-antibody sandwich method for detecting Orientia tsutsugamushi
Source: Front Microbiol. 2025 Jan 15;15:1521015. doi: 10.3389/fmicb.2024.1521015 (PMC11774921; doi:10.3389/fmicb.2024.1521015)
Supplement: Supplementary file 1 [file Data_Sheet_1.docx]

Supplementary material

**Development of a Colloidal Gold Immunochromatographic Assay Utilizing an Optimized Dual-Antibody Sandwich Method with Monoclonal and Polyclonal Antibodies for Detection of *Orientia tsutsugamushi***

**Authors:** Qingyu Lu^1^, Shiyin Yu**^1^,** Sibo Wang^1^**,** Min Cao*^1^，Liuxin Li**^1^** , Miao Xin**^1^**, Weilong Tan^2^,Yong Qi^2^,Yichen Lu*^1^, Xiaohui Xiong^1^

1. College Food Science and Light Industry, Nanjing Tech University, Nanjing 211816, Jiangsu, P. R. China

2. Nanjing Bioengineering (Gene) Technology Center for Medicine, Nanjing 210002, P.R. China

**Corresponding author:** Min Cao, anniecao2001@163.com; Yichen [Lu,yichenlu@njtech.edu.cn](mailto:Lu,yichenlu@njtech.edu.cn)

**Co-authors:** Qingyu Lu,Shiyin Yu and Sibo Wang contributed equally to this work

List of Contents:

**Figure S1. Expression, purification and identification of recombinant protein of BS-717 recombinant protein**

**Figure S2. Identification of monoclonal antibodies against 56 kD recombinnat protein by ELISA**

**Figure S3. Identification of monoclonal antibody targeting conserved 56 kD protein BS 717 by ELISA**

**Figure S4. SDS-PAGE analysis of purified monoclonal antibody 5B3**

**Table S1. Identification of Sj-717 recombinant protein by ELISA**

**Table S2. Identification of mAb 5B3 by ELISA**

**Table S2. QPCR reaction condition**


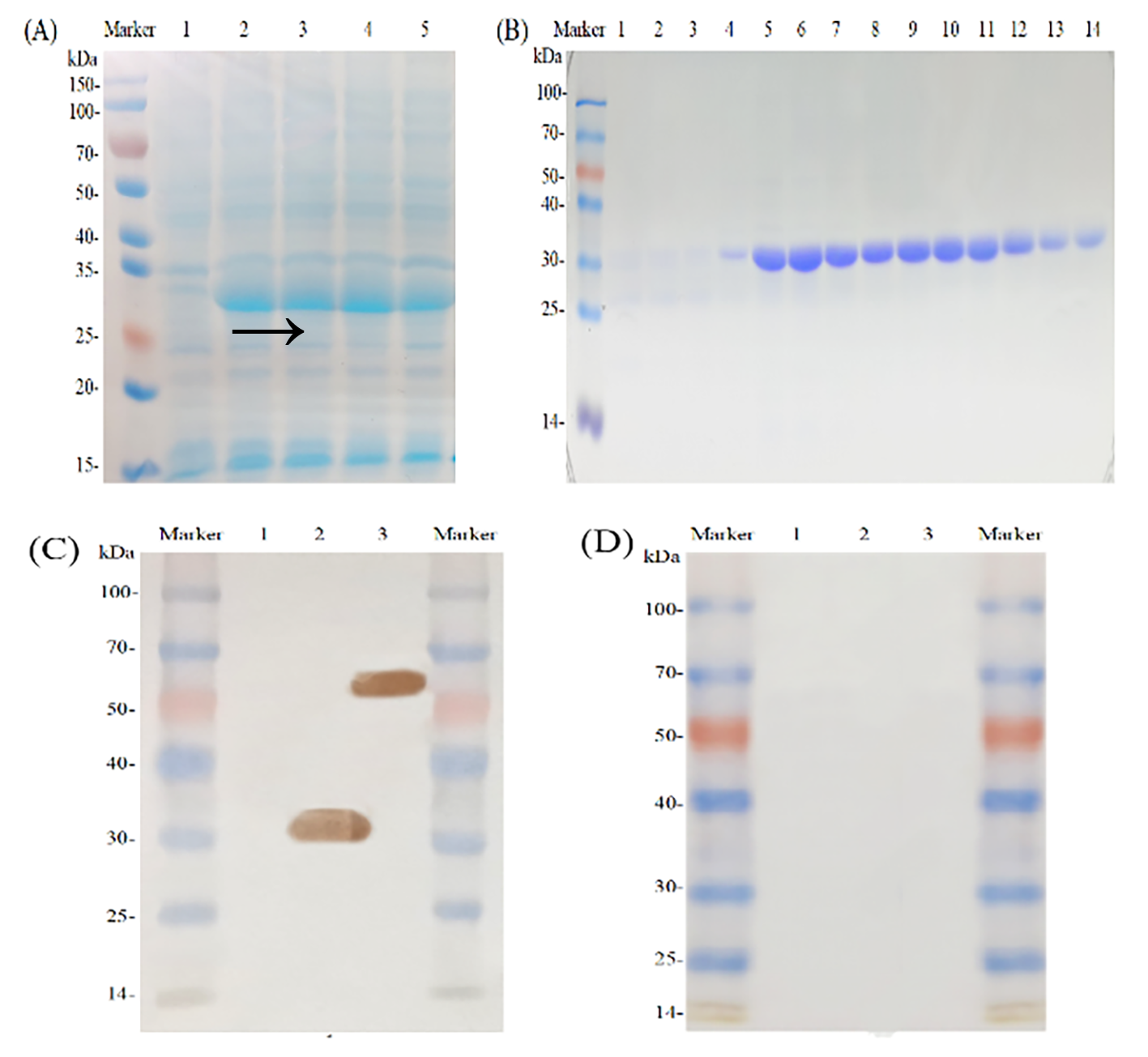


**Figure S1. Expression, purification and identification of recombinant protein of BS-717 recombinant protein** (A) SDS-PAGE analysis of proteins in E. coli before and after induction of the pET-28 expression system. M, Protein Ladder :15~150 kDa. Lane 1, pET-28a control prior to induction, 2-5. Recombinant plasmid pET-SJ-717 after induction. The arrow indicates the recombinant protein in lane 2-5 migrating at the expected position (32 kDa).(B) SDS-PAGE analysis of BS-717 recombinant protein purified by affinity chromatography M, Protein Ladder :14~100 kDa.Lane1-14:different concentrations of imidazole eluent.(C)Western blot identification of recombinant protein BS-717 after purification by Ni affinity chromatography using *O. tsutsugamushi* infects human serums as a probe.M. Protein Ladder 14~100 kDa. Lane 1.pET-28a control, Lane 2, purified BS-717 recombinant protein identified,Lane 3,positive 56 kDa recombinant protein.(D)Western blot identification of recombinant protein BS-717 after purification by Ni affinity chromatography using no patient serum as a probe.M. Protein Ladder 14~100 kDa. Lane 1.pET-28a control, Lane 2, purified BS-717 recombinant protein identified,Lane 3,positive 56 kDa recombinant protein.

The results showed the BS-717 recombinant protein had been successfully expressed and purified and could actively reacted with specific antibodies in *O. tsutsugamushi* infects human serums


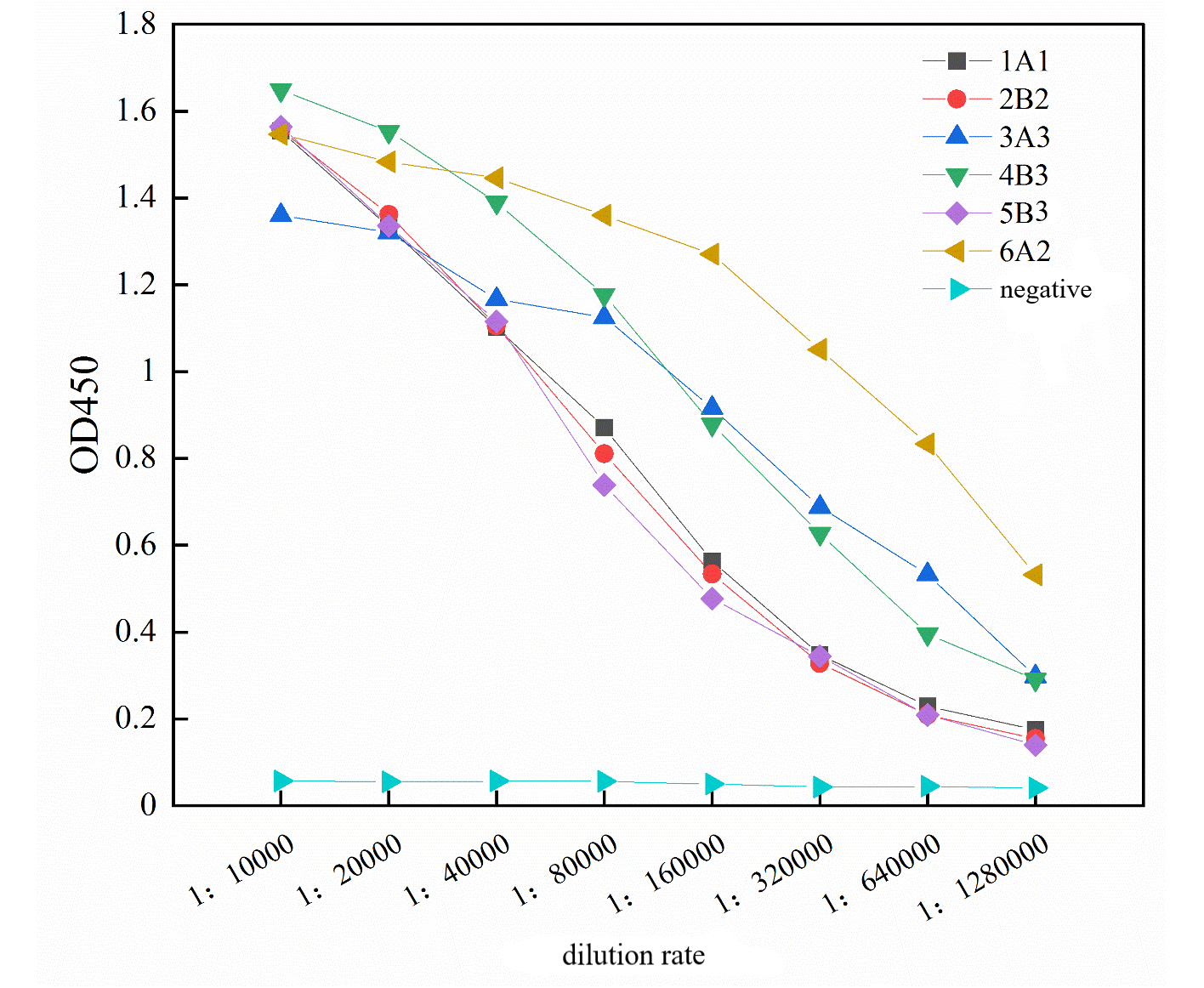


**Figure S2. Identification of monoclonal antibodies against 56 kD recombinnat protein by ELISA**

Mice were immunized with the selected positive single cell lines. Mice ascites was collected and affinity chromatography to purify to obtain the monoclonal antibody. Indirect ELISA showed the purified monoclonal antibody titer.The top lines represent ELISA plots of ascites purified from different single cell lines, the lines below represent Elisa maps of ascites from negative mice.

The results showed that the purified monoclonal antibody had a potency of 1:1280000.


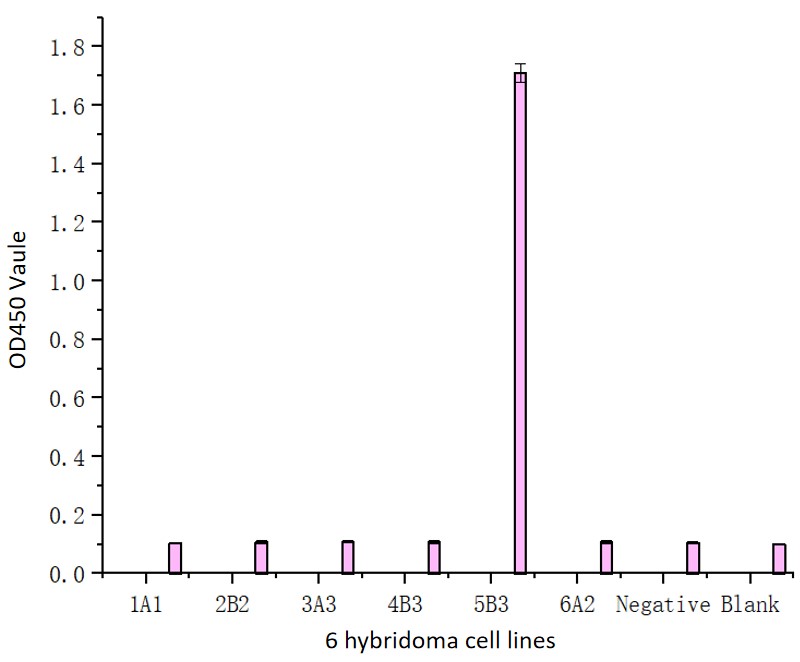


**Figure S3. Identification of monoclonal antibody targeting conserved 56 kD protein BS 717 by ELISA**

Using BS-717 recombinant protein as coated antigen, the supernatants of 6 hybridoma cell lines as primary antibody and goat anti-mouse as secondary antibody, indirect ELISA were performed and OD450 were recorded after the fourth subclone.

The results showed that a hybridoma cell line named 5B3 which could react specifically with BS-717 recombinant protein was selected by indirect ELISA.


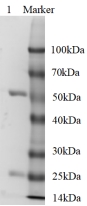


**Figure S4.SDS-PAGE analysis of purified monoclonal antibody 5B3**

Ascites containing 5B3 monoclonal antibody were purified using Protein G column, and the purified sample was analyzed by SDS-PAGE. The results showed that there were obvious bands around 50 kDa and 25 kDa which are the heavy and light chains of antibodies, indicating the successful purification.

|  | Number | OD |  | Number | OD |
| --- | --- | --- | --- | --- | --- |
| Scrub typhus | 1 | 1.231 |  | 1 | 0.089 |
|  | 2 | 0.784 | Schistosome | 2 | 0.084 |
|  | 3 | 1.131 |  | 3 | 0.062 |
|  | 4 | 1.006 |  | 1 | 0.090 |
|  | 5 | 2.074 | Spot fever | 2 | 0.078 |
|  | 6 | 1.034 |  | 3 | 0.065 |
|  | 7 | 0.993 |  | 1 | 0.082 |
|  | 8 | 1.657 | Malaria | 2 | 0.067 |
|  | 9 | 0.864 |  | 3 | 0.068 |
|  | 10 | 0.778 | Hemorrhagic fever | 1 | 0.074 |
|  | 11 | 0.936 |  | 2 | 0.086 |
|  | 12 | 1.863 |  | 3 | 0.077 |
| Negative | 1 | 0.067 |  | 1 | 0.076 |
|  | 2 | 0.080 | Typhoid fever | 2 | 0.087 |
|  | 3 | 0.078 |  | 3 | 0.064 |

**Table S1 ELISA identification of Sj-717 recombinant protein**

BS-717 recombinant protein was coated in the Microtiter plates (96 well) overnight, *O. tsutsugamushi* infects human serums and Peroxidase-conjugated goat anti-human IgG were used as primary and second antibodies respectively, malaria, hemorrhagic fever, typhoid fever, schistosomiasis, spotted fever and normal human serum as a control in each experiment. The results showed that the OD values of the positive and negative serum samples were significantly different, with OD positive/OD negative >2.1, indicating that the BS-717 recombinant protein was active and specifically recognized by the specific antibodies in *O. tsutsugamushi* infects human serums.

| Number | 5B3 | | | Negative mouse serum | | | Blank |
| --- | --- | --- | --- | --- | --- | --- | --- |
|  | 1 | 2 | 3 | 1 | 2 | 3 |  |
| A450 nm | 1.344 | 1.293 | 1.319 | 0.058 | 0.060 | 0.057 | 0.031 |

**Table S2. ELISA identification of mAb 5B3**

5B3 cell line PBS was re-suspended and injected into the abdominal cavity of mice. After a period of time, ascites was collected and purified with Protein G column. After purification, mouse negative serum was used as negative control, and the monoclonal antibody was identified by ELISA. The results showed that the monoclonal antibody was successfully screened and purified.

| 0Procedure | Temperature | Time | Cycle number |
| --- | --- | --- | --- |
| Predegeneration | 95℃ | 3 min | - |
| Denaturation | 95℃ | 15 s | 40cycles |
| Anneal | 58℃ | 20 s |  |
| Extend | 72℃ | 25 s |  |
| Fusion | Instrument preset | | - |

**Table S3. QPCR reaction condition**
